# Supplementary material for: Consensus-based expert recommendations on the management of MPS IVa and VI in Saudi Arabia
Source: Orphanet J Rare Dis. 2024 Jul 17;19:269. doi: 10.1186/s13023-024-03237-3 (PMC11253461; doi:10.1186/s13023-024-03237-3)
Supplement: Supplementary file 2 — Supplementary Material 2 [file 13023_2024_3237_MOESM2_ESM.docx]

**Consensus-based expert recommendations on the management of MPS IVa and VI in Saudi Arabia**

Moeenaldeen AlSayed^1^, Dia Arafa^2^, Huda Al-Khawajha^3^, Manal Afqi^4^, Nouriya Al-Sanna’a^5^, Rawda Sunbul^6^, Maha Faden^7^

^1^Consultant, Department of Medical Genomics, King Faisal Specialist Hospital & Research Centre, Riyadh, Saudi Arabia; ^2^Consultant Pediatrician and Medical Genetics, Maternity and Children Hospital, Makkah, Saudi Arabia; ^3^Consultant Pediatrician & Medical Genetics, Maternity and Children Hospital, Al-Ahsa, Saudi Arabia; ^4^Consultant Pediatrician, Clinical Genetics and Metabolic Disorders, Maternity and Children Hospital, Madinah, Saudi Arabia; ^5^Clinical Geneticist, Johns Hopkins Aramco Healthcare, Dhahran, Saudi Arabia; ^6^Consultant Pediatrician and Medical Genetics, Qatif Central Hospital, Qatif, Saudi Arabia; ^7^Consultant Pediatrician, Clinical Genetics – Metabolic and Skeletal Dysplasia, Genetic Unit, Maternity and Children Hospital, King Saud Medical City, Riyadh, Saudi Arabia

**Corresponding author:**

Moeenaldeen AlSayed

Consultant, Department of Medical Genomics, King Faisal Specialist Hospital & Research Centre, Riyadh, Saudi Arabia

E-mail address: [moeen@kfshrc.edu.sa](mailto:moeen@kfshrc.edu.sa)

**Abstract**

Background

Mucopolysaccharidosis type IVa (Morquio A syndrome) and mucopolysaccharidosis type VI (Maroteaux-Lamy syndrome) are rare inherited lysosomal storage diseases associated with significant functional impairment and a wide spectrum of debilitating clinical manifestations. These conditions are thought to have higher-than-average prevalence rates in Saudi Arabia due to high rates of consanguineous marriage in the country. There are several unmet needs associated with the management of these diseases in Saudi Arabia.

Main Body

The aim of this manuscript is to contextualize unmet management needs and provide recommendations to optimize diagnosis, multidisciplinary care delivery, and local data generation in this disease area. An expert panel was assembled comprising seven consultant geneticists from across Saudi Arabia. The Delphi methodology was used to obtain a consensus on statements relating to several aspects of mucopolysaccharidosis types IVa and VI. A consensus was reached for all statements by means of an online, anonymized voting system. The consensus statements pertain to screening and diagnosis, management approaches, including recommendations pertaining to enzyme replacement therapy, and local data generation.

Conclusion

The consensus statements presented provide specific recommendations to improve diagnostic and treatment approaches, promote multidisciplinary care and data sharing, and optimize the overall management of these rare inherited diseases in Saudi Arabia.

**Keywords**

MPS; Morquio A syndrome; Maroteaux-Lamy syndrome; lysosomal storage disease; clinical genetics; metabolic disease; multidisciplinary care; enzyme replacement therapy

**Background**

The mucopolysaccharidoses (MPS) are a rare group of inherited lysosomal storage diseases (LSDs) characterized by the deficiency of enzymes required to degrade glycosaminoglycans (GAGs), resulting in a wide spectrum of clinical manifestations (1). MPS IVa (Morquio A syndrome) is caused by pathogenic mutations in *GALNS* and is associated with deficiency of the enzyme N-acetylgalactosamine-6-sulfatase (GALNS), resulting in pathological accumulation of keratan sulfate and chondroitin-6-sulfate (2). MPS IVa is marked by impairment of cartilage and bone development, leading to systemic skeletal dysplasia. Clinical features include short neck and trunk dwarfism, atlantoaxial subluxation, pectus carinatum, kyphoscoliosis, and gait difficulty. MPS IVa is also associated with cardiac and pulmonary complications, hearing and vision loss, fatigue, and dental abnormalities (3-8).

MPS VI (Maroteaux-Lamy syndrome) is caused by pathogenic mutations in *ARSB*, resulting in deficiency of the enzyme arylsulfatase B (ARSB) and lysosomal deposition of dermatan sulfate and chondroitin-4-sulfate (9). Clinical symptoms of MPS VI include coarse facial features, umbilical hernia, joint contractures, and hepatosplenomegaly (8). MPS VI is also associated with growth impairment, neurological symptoms, cardiac and pulmonary complications, skeletal and dental abnormalities, and vision and hearing impairment (10-13). However, both MPS IVa and MPS VI are thought to have minimal impacts on cognitive function, distinguishing them from other MPS subtypes (8, 14, 15).

MPS IVa and VI have a diverse spectrum of clinical symptoms affecting multiple organ systems and variable ages of presentation and progression rates (10, 16, 17). In both disease subtypes, classical and attenuated phenotypes have been characterized, with the latter referring to a less severe form of the respective condition associated with residual enzyme activity, slower disease progression, and later onset of symptoms (4, 9, 18). As clinical presentations vary considerably among patients for both disease subtypes, timely and accurate diagnosis represents a significant challenge. Moreover, early treatment initiation and comprehensive management by a multidisciplinary healthcare team are crucial to address the specific needs of affected individuals and provide integrated care for optimized patient outcomes (10, 17).

The incidence of MPS IVa is estimated as 1 in 201,000 live births but is thought to vary widely among different populations (18). The birth prevalence of MPS VI is estimated to be between 1 in 43,261 and 1 in 1,505,160 (19-21). The prevalence of both subtypes is thought to be higher in Saudi Arabia due to high rates of consanguinity in the country and relatively high homogeneity of the gene pool (22). An epidemiological study examining the incidence of inborn errors of metabolism in the eastern province of Saudi Arabia from 1983 to 2008 reported that the prevalence of MPS IV (a and b) and MPS VI per 100,000 live births is 3.62 and 7.85, respectively (23). However, the true prevalence of these conditions cannot be accurately determined due to the lack of a robust national screening program or large-scale screening programs. There is currently a significant unmet need to integrate large-scale screening initiatives such as premarital screening for primary prevention and newborn screening for secondary prevention to optimize the detection of MPS IVa and VI in Saudi Arabia (22, 23).

Accurate and timely diagnosis of MPS IVa and VI is crucial for optimizing patient outcomes and quality of life. Presentations of these conditions are diverse, with a large number of potential causative mutations and varied phenotypic spectrums with multisystemic manifestations (10, 16, 17). Diagnostic approaches for these rare LSDs involve clinical evaluation, radiographic investigation, biochemical testing, and genetic analysis (24). Elevated urinary levels of GAGs can be detected via urine tests; however, these findings in isolation are not sufficient to establish a diagnosis (25). Assessment of enzyme activity levels is a crucial component of the diagnostic processes in MPS IVa and VI and are usually conducted in leukocytes or cultured fibroblasts (4). Furthermore, molecular analysis can confirm a diagnosis by identifying mutations in the genes that encode enzymes involved in GAG degradation (i.e., *GALNS* and *ARSB*). While generally more costly to perform than other tests, molecular analysis can be used as a standalone diagnostic approach and supports carrier testing, prenatal testing, and genetic counseling for families in which pathogenic variants are identified (25).

Dry blood spot (DBS) testing represents a significant advancement in the diagnosis of MPS IVa and VI due to the ease of collection, storage, and transport of samples (26). DBS samples can be used to perform multiple tests of enzyme activity as well as molecular analysis. Liquid chromatography/tandem mass spectrometry (LC-MS/MS) to identify specific enzyme deficiencies in blood or urine has also emerged as a valuable tool in diagnosis and monitoring. Novel LC-MS/MS assays are more effective and accurate than traditional fluorometric methods and are therefore ideal for integration within potential newborn screening programs for MPS IVa and VI (23, 27). These rare diseases are challenging to diagnose, and Saudi Arabia faces additional challenges that further complicate diagnosis, such as inadequate disease awareness, a lack of essential laboratory facilities and resources in some regions, and sociocultural issues such as stigmatization leading to denial, isolation, and non-disclosure of the conditions (22).

In MPS IVa and VI, immediate treatment initiation upon confirmation of diagnosis is crucial as early intervention may minimize risks of irreversible deterioration (10, 17, 28). Enzyme replacement therapy (ERT) with elosulfase alfa for MPS IVa and galsulfase for MPS VI are approved targeted treatment options with demonstrated efficacy in symptom alleviation, improved mobility, pulmonary benefits, and enhanced quality of life (4, 22, 29). However, ERT requires weekly infusions of costly recombinant enzymes and demonstrates limited efficacy for several MPS features, such as ocular and skeletal abnormalities as well as established cardiac disease (30-32). This may in part be attributed to untimely intervention and incorrect dosing (33, 34). Allogeneic hematopoietic stem cell transplantation (HSCT) has also been demonstrated to be a viable treatment option for select patients with MPS but is considered ineffective for MPS IVa (35, 36). Several other potential therapies are also under investigation in clinical and preclinical studies, including gene therapies, next-generation ERTs (e.g., PEGylated enzymes and high-dose ERTs), novel enzyme formulations (e.g., fusion proteins and optimized enzymes), and small molecule, chaperone, and substrate reduction therapies (37-43). Furthermore, combination therapies of ERT with gene therapy or small molecule drugs are also under investigation (44, 45). Non-pharmacological interventions include physiotherapy, patient education, and physical activity (22). For optimized patient outcomes, it is crucial that patients with MPS IVa and MPS VI undergo frequent follow-up assessments. Integrated and comprehensive care should be delivered by a multidisciplinary management team including a diverse range of medical subspecialties and tailored to the specific needs of each patient. There are currently several unmet needs pertaining to the management of MPS IVa and VI in Saudi Arabia that warrant special consideration, including a general paucity of specialized adult geneticists, eligibility issues for care coverage, the lack of established national management guidelines, and other logistical challenges (22).

The landscape of MPS IVa and VI is highly nuanced in Saudi Arabia, with several unmet needs that hinder the delivery of optimal care. The fragmentation of the national healthcare system and lack of a national data registry for MPS are also significant detriments to effective management of these rare and debilitating LSDs. Though management guidelines in MPS IVa and VI have previously been published, this is the first publication to delineate recommendations specific to Saudi Arabia, with consideration of the national healthcare infrastructure and patient population (10, 17, 28). This position paper aims to contextualize local unmet needs and propose potential solutions to improve the quality, consistency, and accessibility of care in Saudi Arabia, with the overarching goal of enhancing patient outcomes and quality of life in MPS IVa and VI.

**Main Text**

Methods

*Assembly of Expert Panel*

An expert panel was established comprising seven consultant geneticists with clinical experience in the management of MPS IVa and VI from across Saudi Arabia. The panel convened on September 28, 2023, to discuss the landscape of MPS IVa and VI in the country in the context of screening and diagnosis, management options, current unmet needs, and opportunities to optimize care.

*Literature Review*

A literature review was conducted using the PubMed database to identify pertinent treatment guidelines, randomized controlled trials, and observational studies to allow for the generation of evidence-based consensus statements.

*Survey*

The expert panel completed a 19-question survey via an online platform, which aimed to gather insights into their clinical experience managing MPS IVa and VI, with special focus on patient characteristics, unmet management needs, and treatment selection considerations.

*Development of Consensus Statements*

Consensus statements relating to clinical aspects of MPS IVa and VI were jointly developed by the team of authors, who together also represent the expert voting panel. The developed statements were largely congruent with international guidelines; however, evidence-based recommendations were made to align them closely with the local clinical landscape, with consideration of the nuances of the specific patient population and national healthcare infrastructure. The expert panel also reviewed relevant published findings from various randomized controlled trials and observational studies during the development of the consensus statements. Each consensus statement was critically evaluated and modified as required by all members of the panel. The Delphi technique was used to reach a consensus on all statements, with panel members anonymously voting on each statement. Voting options were “strongly agree”, ‘”agree”, “neither agree nor disagree”, “disagree”, “strongly disagree”, and “abstain/unable to answer”. A consensus was considered when ≥80% of experts chose to “strongly agree” or “agree” with a particular statement.

Results

The expert panel reached a consensus on six statements pertaining to screening and diagnosis. These highlight the need to improve the availability of diagnostic and monitoring tests and genetic counseling and to raise awareness of LSDs among healthcare professionals of different subspecialties. The panel also recommends initiating a newborn screening pilot program to determine the incidences of MPS IVa and MPS VI in the country. These statements are listed in Table 1.

The panel reached a consensus on 13 statements pertaining to the management of MPS IVa and MPS VI. These statements cover treatment initiation, ERT home infusion programs, multidisciplinary care delivery, individualized care, and the establishment of a national reimbursement program for rare diseases in Saudi Arabia. These statements are listed in Table 2.

The panel reached a consensus on two statements pertaining to local data generation. These emphasize the need for local MPS IVa and MPS VI data generation, data sharing, and the establishment of a robust national registry for LSDs. These statements are listed in Table 3.

Discussion

*MPS IVa and MPS VI in Saudi Arabia*

MPS IVa and VI represent significant health challenges in Saudi Arabia for affected individuals, their families, and the wider healthcare system. Both conditions have a diverse phenotypic spectrum and are associated with significant debilitation and impairment (10, 24). Moreover, the landscape of MPS in Saudi Arabia is nuanced, with unique challenges that warrant specific considerations.

*Screening and Diagnosis of MPS IVa and VI*

Clinical presentations of MPS IVa and VI are heterogenous with non-specific, multisystemic symptoms that can vary in severity among patients (10, 16-19). Patients may present with an attenuated phenotype, characterized by residual enzyme activity and slower disease progression compared with classical MPS IVa and VI phenotypes (4, 9, 18). This variability coupled with inadequate disease awareness frequently hinders timely diagnosis, which is especially critical in cases of rapidly progressing skeletal dysplasia (4, 24). Early recognition and treatment initiation can improve patient quality of life by delaying the development of irreversible pathologies (24, 33). Additionally, many typical features of MPS IVa and VI are shared among other MPS subtypes, other LSDs such as multiple sulfatase deficiency, mucolipidosis, Fabry disease, hereditary skeletal dysplasias, and some metabolic and connective tissue disorders (19, 24, 46-48). Accurate diagnosis is therefore essential to ensure that patients receive appropriate care and disease-specific treatment (24).

Biochemical diagnostic approaches for MPS encompass urine testing and enzyme activity analysis. Urine tests allow for non-invasive assessment of abnormalities in GAG excretion. The accumulation of GAGs is a hallmark of MPS, and urine testing can provide a useful initial indication, guiding further diagnostic evaluation. However, urine testing in isolation is insufficient to establish a diagnosis, and in cases of clinical suspicion of MPS IVa, it is strongly recommended to proceed to enzyme activity analysis even if urinary GAG levels appear normal (25, 49). Enzyme activity analysis is a vital diagnostic approach that involves assessing the activity of specific enzymes, such as GALNS for suspected MPS IVa and ARSB for suspected MPS VI (24, 25, 49). Reduced enzyme activity in leukocytes or cultured fibroblasts are key diagnostic markers for these disorders. Enzyme activity assays can help confirm a diagnosis and distinguish between different MPS subtypes as well as other disorders, providing critical insights to guide management decisions. Fluorometric techniques are most commonly used for enzyme assays, though recent LC-MS/MS technologies allow for more precise and sensitive quantification of enzyme levels (4, 27, 50).

Molecular genetic analysis can be used as a confirmatory diagnostic approach when a pathogenic variant is present on each allele of *GALNS* or *ARSB* for MPS IVa and VI, respectively. A large number of pathogenic mutations in these genes have been characterized, including missense and nonsense mutations, rearrangements, insertions, and deletions (25, 49, 51, 52). Molecular analysis can be conducted using leukocytes from whole blood and DNA from saliva samples, and can facilitate phenotype prediction, carrier testing, prenatal testing, and genetic counseling (24, 46, 53). Specific mutations have been demonstrated to correlate with residual enzyme activity in MPS IVa and this can be used to predict clinical severity (4, 51, 54-57). Molecular genetic analysis can also be used to discriminate between pseudodeficiency, carrier status, and normal status in cases wherein there is low enzyme activity and normal urinary GAG levels (58). Molecular analysis is a precise and essential component of the diagnostic process for MPS IVa and MPS VI, enabling appropriate care and management for affected individuals when considered together with clinical and biochemical findings. However, molecular analysis is generally more costly than other diagnostic tests and may uncover novel variants of unknown significance.

DBS-based assays allow for multiple enzyme activity tests as well as molecular analysis and offer several logistical advantages in terms of sample collection, storage, and transport (24). While these assays are generally sensitive and specific if performed with appropriate controls, they are insufficient to confirm a diagnosis of MPS IVa or VI in isolation due to the limited number of cells present in the sample and the risk of a false positive result due to sample degradation (46, 49). Confirming results obtained from DBS assays via enzyme activity analysis in other tissues and/or molecular analysis is strongly recommended. Furthermore, a combination of clinical findings and biochemical/molecular genetic test results is crucial to ensure an accurate diagnosis in all cases of suspected MPS IVa and VI.

In Saudi Arabia, there is a significant disparity in access to specialized laboratory tests. Urinary GAG assays and enzyme activity tests are unavailable in remote areas where some centers lack necessary laboratory facilities, trained personnel, or logistical capabilities for send-out testing. These tests are mostly available in established tertiary centers as send-out tests. The shortage of specialized staff in some regions represents another challenge in carrying out critical laboratory tests and interpreting results accurately. Therefore, there is an urgent need to improve the accessibility and availability of biochemical diagnostic and monitoring tests for MPS IVa and VI across treatment centers in Saudi Arabia. Furthermore, there was a consensus among the expert voting panel that molecular testing should be made available to all individuals with suspected MPS IVa and VI to identify the causative pathogenic variant. This should be coupled with genetic counseling, which should also be available and easily accessible.

*Premarital Screening*

The expert panel highlighted that premarital screening initiatives should be reinforced among select families with a history of MPS IVa and/or MPS VI in Saudi Arabia. This proactive approach to primary prevention can have a profound impact on reducing the occurrence of these rare genetic disorders within affected communities. Premarital screening allows prospective couples to assess their genetic compatibility and the risk of passing on these inheritable conditions to their offspring. By identifying carriers of MPS IVa and MPS VI, individuals can make informed decisions regarding family planning, consider genetic counseling, and explore options such as prenatal testing or assisted reproductive technologies if appropriate (22). Premarital screening initiatives can empower families with essential genetic information and contribute to the prevention and management of MPS IVa and VI.

*Newborn Screening for MPS IVa and MPS VI*

Newborn screening has the potential to transform the landscape of MPS detection in Saudi Arabia. Establishing a correct diagnosis for MPS IVa and VI usually takes several years (46). In most cases of MPS, patients are asymptomatic at birth and subsequently experience the onset of clinical symptoms (50). Moreover, patient outcomes for some MPS subtypes are significantly improved with early detection and timely intervention prior to the development of irreversible pathologies and debilitating manifestations (53, 59). It is therefore recommended that pilot newborn screening initiatives are implemented in Saudi Arabia to assess impacts on long-term outcomes of MPS IVa, MPS VI, and other LSDs. It has been demonstrated that novel LC-MS/MS methods are more precise than traditional fluorometric assays and can support multiplex newborn screening (27). Additionally, LC-MS/MS can be integrated with DBS testing for newborn screening (46). DBS represents the preferred sample for this purpose as it offers several logistical advantages (60).

*Raising Awareness of MPS IVa and VI*

There was a consensus among the expert voting panel that there is a need to raise awareness of different LSDs, including MPS IVa and VI, among healthcare professionals across various subspecialties in Saudi Arabia. Improving awareness can raise clinical suspicion of these conditions when appropriate, potentially allowing for the detection of cases that would otherwise be undiagnosed (22). Establishing clinical suspicion when appropriate is critical, as initial signs and symptoms may vary among patients and the presence or absence of any particular feature is insufficient to confirm or rule out MPS (61). Patients may initially be seen by family physicians in primary healthcare, surgeons, or ear, nose and throat (ENT), pediatric, orthopedic, ophthalmologic, cardiac, or rheumatologic specialists, who may not have familiarity with MPS presentations, particularly attenuated phenotypes (24, 49, 62). Increased awareness of MPS among these specialties is required to facilitate timely referral to a clinical geneticist or metabolic specialist. Potential initiatives to improve awareness of different LSDs include conducting periodic lectures and workshops, disseminating educational material to medical staff, and promoting bilateral communication between different subspecialties (22).

Furthermore, there are sociocultural issues associated with rare, inherited diseases in Saudi Arabia. Patients and families often face stigmatization and isolation, and this can lead to denial and non-disclosure of these diseases. These sociocultural challenges can also have a negative impact on treatment adherence and psychological well-being (22). To combat these issues, the expert panel recommends that community-based initiatives are conducted, such as general awareness campaigns with specific messaging, and the provision of tailored education and psychological support for patients and their families.

*ERT for MPS IVa and MPS VI*

Early intervention is key to optimizing treatment outcomes in MPS IVa and VI (10, 17, 28, 33, 53). As such, it is recommended that treatment is initiated immediately after confirmation of a diagnosis and family counseling to discuss treatment options and expected outcomes. This recommendation is in alignment with international guidelines for the management of MPS IVa and VI (28, 63). ERT with elosulfase alfa, a recombinant form of GALNS, is the primary disease-specific treatment option for MPS IVa. The aim of treatment with elosulfase alfa is to prevent lysosomal accumulation of keratan sulfate and chondroitin-6-sulfate and thus prevent disease manifestations. In a phase III, randomized, placebo-controlled study of the efficacy and safety of elosulfase alfa for MPS IVa, patients who received 2.0 mg/kg/week over 24 weeks experienced reductions in urinary GAGs and improvements in endurance related to enhanced respiratory function (64). Other studies have demonstrated minor benefits in some other domains such as daily activities and motor skills (65-67). These findings are indicative of partial recovery of functionality following treatment with elosulfase alfa for MPS IVa (63). Elosulfase alfa also demonstrates an acceptable safety profile (68). While HSCT may be a viable treatment option for select patients with MPS, it is thought to be ineffective in MPS IVa (35, 36). As per a survey conducted among members of the expert voting panel, the majority of respondents “strongly agreed” with the statement “HSCT is ineffective in MPS IVa and is not recommended for these patients” (Fig. 1) (22).

*Figure 1: Perceptions of effectiveness of HSCT in treating MPS IVa among the expert panel*

In addition to HSCT, ERT with galsulfase is the primary disease-specific treatment option for MPS VI. Treatment with galsulfase, a recombinant form of ARSB, aims to prevent lysosomal accumulation of dermatan sulfate and chondroitin-4-sulfate and thus prevent MPS VI disease manifestations (28). Several studies have demonstrated reduced urinary GAGs and improved endurance and pulmonary function in patients receiving ERT with galsulfase (29, 69-72). It is also thought that galsulfase is associated with improved growth and mobility, delayed progression of cardiac abnormalities and bone disease, and improved quality of life in patients with MPS VI (73-77). It is noteworthy that several adverse safety events have been reported in clinical trials involving galsulfase, including fever, nausea, vomiting, headache, respiratory distress, and infusion-site reactions (28).

ERT outcomes in MPS IVa and VI are significantly improved when treatment is initiated early in life (10, 17, 28, 63). There was a consensus among the expert panel that ERT (elosulfase alfa for MPS IVa and galsulfase for MPS VI) should be accessible to all eligible patients in Saudi Arabia. ERT represents a treatment option in the management of these rare LSDs and can restore partial function, slow the progression of disease manifestations, and improve patient quality of life in MPS IVa and VI (28, 63). Response to treatment is variable, with less severely affected patients more likely to benefit from ERT. To maximize its benefits, it is crucial that ERT is readily available to all eligible patients across the country. In all cases, treatment plans in MPS IVa and VI should be highly individualized. Informed decision-making should be promoted with extensive patient/family counseling in close consultation with a genetic/metabolic specialist to address the specific needs and clinical profile of each patient. Moreover, it is recommended that formal standards of care for MPS IVa and VI are established in Saudi Arabia, encompassing clear guidelines that specify when ERT is indicated as a viable treatment option and when treatment cessation is warranted (22).

*ERT Home Infusion Programs*

It is recommended that ERT home infusion programs are established along with clear selection criteria and guidelines to improve adherence among patients and combat sociocultural and logistical challenges (22). Home infusion programs may enhance patient adherence by reducing the burden of frequent hospital visits and improving self-perceived quality of life (78, 79). As per local experience, home infusion programs clearly improve adherence, with some patients demonstrating full treatment compliance over a long-term period. Moreover, these initiatives can help address sociocultural issues such as stigma or apprehension related to healthcare facilities, which may discourage individuals from pursuing treatment. By promoting a patient-centric approach and addressing cultural and logistical nuances, ERT home infusion programs can significantly improve treatment adherence and thus enhance clinical outcomes for individuals with MPS IVa and MPS VI in Saudi Arabia. As per a survey conducted among the expert panel, the majority of respondents “strongly agreed” that the provision of ERT home infusion solutions can improve adherence and health outcomes for patients with MPS IVa and MPS VI in the country (22).

*Multidisciplinary Care Approach for MPS IVa and MPS VI*

Promoting multidisciplinary care for MPS IVa and VI in Saudi Arabia is crucial to enhance patient quality of life and overall outcomes. The utilization of multidisciplinary care teams that encompass metabolic specialists, surgeons, nurses, physiotherapists, occupational therapists, psychologists, audiologists, and speech pathologists can significantly alleviate the burden of these diseases on individuals and on the healthcare system via improved health outcomes and optimized resourcing. To address the multisystemic manifestations of MPS IVa and VI, care teams should also engage pulmonology, dental, orthopedic, pediatric, ENT, ophthalmology, cardiology, anesthesiology, and neurology specialists as required (22). Patients should also have access to extensive psychological support and education to address issues such as stigmatization and isolation as part of a comprehensive care approach for MPS IVa and MPS VI in Saudi Arabia. Adopting a holistic, patient-centric approach goes beyond addressing the medical aspects of MPS IVa and MPS VI and encompasses the broader spectrum of physical, psychological, and social needs of affected individuals (28, 63). An integrated model of care can be instrumental in minimizing complications by addressing multisystemic manifestations and improving psychological well-being and quality of life for patients (17). This recommendation is in alignment with recognized international management guidelines for MPS IVa and VI (17, 28, 63).

However, there are several challenges associated with adopting multidisciplinary care in Saudi Arabia. Firstly, the national healthcare system is fragmented and there is a lack of coordination between centers. Different centers also utilize different criteria for patient eligibility for therapy. Additionally, there is a lack of specialized adult geneticists in the country. Conducting initiatives to increase interest in adult genetics is therefore warranted. Other challenges pertaining to the implementation of multidisciplinary care are frequent staff turnover and insufficient experience in managing complex symptoms of MPS among other subspecialties like physiotherapy and orthopedics (22). Currently, most centers in Saudi Arabia lack multidisciplinary care teams for MPS IVa and VI; however, the implementation of multidisciplinary care is in progress at some tertiary care centers.

*Individualized Care for MPS IVa and MPS VI*

There was a consensus among the expert panel that treatment plans for patients with MPS IVa and MPS VI should be highly individualized. Individualized care for these disorders is essential due to the diverse spectrum of multisystemic symptoms and their varying severities among patients. These disorders can manifest with skeletal, cardiac, respiratory, ophthalmological, gastrointestinal, and dental abnormalities, and thus necessitate tailored interventions (10, 16-19). By collaborating with different specialists within multidisciplinary healthcare teams, genetic/metabolic specialists can develop individualized treatment plans to optimize care and improve patient outcomes in MPS IVa and MPS VI.

*Continuity of Care*

There are several major challenges associated with transitioning patients from pediatric to adult care in Saudi Arabia. Firstly, fragmentation of the healthcare system and the lack of coordination between different centers represents a challenge. In many cases, patient data is unavailable to the new treatment center due to inadequate data sharing and collaboration. Additionally, standalone maternity and children’s hospitals do not offer adult services. Hospitals catering to adults are entirely separate facilities, and there is insufficient collaboration between pediatric and adult centers. Furthermore, there is a lack of a clear referral system and guidelines when transitioning patients from pediatric to adult care, leading to confusion and inconsistent practices. In most cases, transitions in care are dependent on personal communication among physicians. Furthermore, there is a paucity of adult metabolic specialists and geneticists in the country. Consequently, adult patients with MPS IVa and MPS VI are seen by internists under the supervision of pediatric geneticists in some centers. Finally, there may also be logistical challenges that hinder care, such as transportation issues. This is especially relevant to families including pediatric and adult patients with MPS, which are usually separated to receive care at two or more centers (22). Coordinated strategies are required to facilitate the transition of patients with MPS IVa and MPS VI from pediatric to adult care, as continuity of care represents a significant bottleneck in the context of optimal management for these diseases.

*Establishing National Centers of Excellence*

There is a need to establish national centers of excellence for the management of LSDs in Saudi Arabia to ensure the delivery of standardized, comprehensive care. These specialized centers would serve as hubs of expertise, bringing together multidisciplinary teams including genetic/metabolic specialists and allied healthcare professionals with experience in the management of LSDs. By centralizing expertise and resources, these centers can standardize the diagnostic process and management of LSDs, ensuring that patients receive the highest quality of care (22). Moreover, these centers can facilitate research, education, and awareness initiatives, further advancing the understanding and management of these complex disorders. The establishment of national centers of excellence would represent a pivotal commitment to enhancing the outcomes and quality of life of individuals affected by LSDs in Saudi Arabia.

*Landscape of MPS IVa and VI Management in Saudi Arabia*

It is recommended that healthcare staff involved in the delivery of care for MPS IVa and VI undergo periodic training and education to stay updated on the latest advancements in emerging therapies and management guidelines. Furthermore, it is crucial to establish robust systems for monitoring and efficiently integrating approved advanced therapies within the healthcare system of Saudi Arabia. There are several investigational agents for MPS IVa and VI currently being studied in preclinical and clinical trials, including gene therapies, next-generation ERTs, small molecule drugs, and substrate reduction therapies (37-43). This ongoing research holds promise for expanding treatment options and further improving the outlook for individuals affected by these disorders.

Furthermore, it is recommended that regional experts in collaboration with the appropriate national health authorities publish updated guidelines pertaining to the management of MPS IVa and VI. The guidelines should include standards of care, protocols for diagnosis and treatment, and directives pertaining to the establishment of multidisciplinary care teams and centers of excellence (22). There is also a pressing need to establish a national reimbursement program for rare diseases in Saudi Arabia to improve access to crucial medications and care. This program should also address the expatriate subpopulation. Currently, there are disparities in insurance coverage and access to care between expatriate and local subpopulations in Saudi Arabia. While nationals are fully covered by governmental medical insurance policies, expatriates must self-pay or utilize private insurers and are unable to access public healthcare. A large proportion of the expatriate subpopulation in Saudi Arabia is unable to afford premium insurance, and most genetic diseases are not covered in the vast majority of private insurance policies (22). Due to the high costs of ERTs, eligible patients with MPS IVa and MPS VI may therefore be unable to access the care they need. A survey conducted among the panel of consultant geneticists demonstrated that expatriates represent less than 10% of the MPS IVa and VI patient population for the majority of expert panel members (Fig. 2) (22).

*Figure 2: Proportion of expatriates among MPS IVa and MPS VI patient population*

*Data Generation and Reporting*

There is a pressing unmet need for local MPS IVa and MPS VI data generation and the establishment of a centralized, robust national registry for LSDs. Comprehensive and population-specific epidemiological, clinical, and genetic data are essential to understand the prevalence and clinical spectrum of MPS IVa and VI specific to the country. Existing treatment centers and novel centers of excellence should actively engage in data collection and research initiatives, potentially in collaboration with dedicated research institutions and universities. To achieve this, treatment centers in the region should employ data teams with knowledge of disease aspects consisting of data coordinators and separate teams for data entry, data mining, and quality management (22).

However, there are significant regulatory challenges associated with data collection and reporting in Saudi Arabia. Local regulations pertaining to medical data are highly restrictive and mostly prohibit data sharing (22). Furthermore, physical paper records have not been integrated with newly implemented electronic record systems. In addition to regulatory hurdles, data generation is impeded by consent and privacy considerations, cross-institutional challenges, financial constraints, and issues relating to data quality and standardization (22). Data generation initiatives and the prospective establishment of a national data registry for LSDs will therefore require alignment with the local health ministry.

Data generation is especially complicated in the MPS IVa and MPS VI disease areas. As these are rare diseases with small patient populations, there is a scarcity of available data. Furthermore, the heterogeneity of disease manifestations and lack of established outcome measures make it difficult to accurately describe the natural history and progression of MPS IVa and VI. Therefore, there is a critical need for enhanced collaboration and data sharing among MPS IVa and VI treatment centers in Saudi Arabia (22). Promoting data sharing and initiatives to foster collaboration between treatment centers would also optimize the transition of patients from pediatric to adult care.

**Conclusion**

There are currently significant challenges in the management of MPS IVa and MPS VI in Saudi Arabia. The panel of consultant geneticists reached a consensus on statements pertaining to screening and diagnosis, enzyme replacement therapy, multidisciplinary care, and local data generation for these rare inherited diseases.

**List of abbreviations**

| ARSB | arylsulfatase B |
| --- | --- |
| DBS | dry blood spot |
| ENT | ear, nose, and throat |
| ERT | enzyme replacement therapy |
| GAG | glycosaminoglycan |
| GALNS | N-acetylgalactosamine-6-sulfatase |
| HSCT | hematopoietic stem cell transplantation |
| LC-MS/MS | liquid chromatography/tandem mass spectrometry |
| LSD | lysosomal storage disease |
| MPS | mucopolysaccharidoses |

**Declarations**

Ethics Approval and Consent to Participate

This article is based on previously conducted studies and does not contain any new studies with human participants or animals performed by any of the authors.

Consent for Publication

Not applicable.

Availability of Data and Materials

Not applicable.

Competing Interests

Moeenaldeen AlSayed, Dia Arafa, Huda Al-Khawajha, Manal Afqi, Nouriya Al-Sanna’a, Rawda Sunbul, and Maha Faden received honoraria fees from BioMarin Pharmaceutical Inc. as advisory board members.

Funding

This work, including publication and open access fees, was funded by BioMarin Pharmaceutical Inc., Dubai, UAE.

Author Contributions

Moeenaldeen AlSayed, Dia Arafa, Huda Al-Khawajha, Manal Afqi, Nouriya Al-Sanna’a, Rawda Sunbul, and Maha Faden contributed to the development of this manuscript. MS developed consensus statements and prepared the content of the paper. MS, DA, HK, MA, NS, RS, and MF discussed and amended the consensus statements and reviewed the content of the paper. All authors approved the final manuscript.

Acknowledgements

Medical writing support was provided by Luqman Khan of Connect Communications, Dubai, UAE and funded by BioMarin Pharmaceutical Inc., Dubai, UAE. BioMarin Pharmaceutical Inc., Dubai, UAE sponsored the advisory board and manuscript development but did not influence the expert opinions reflected in this manuscript nor provided any scientific review.

**References**

1. Wraith JE. Chapter 102 - Mucopolysaccharidoses. In: Rimoin D, Pyeritz R, Korf B, editors. Emery and Rimoin's Principles and Practice of Medical Genetics (Sixth Edition). Oxford: Academic Press; 2013. p. 1-40.

2. Regier DS, Oetgen M, Tanpaiboon P. Mucopolysaccharidosis Type IVA. In: Adam MP, Mirzaa GM, Pagon RA, Wallace SE, Bean LJH, Gripp KW, et al., editors. GeneReviews((R)). Seattle (WA)1993.

3. Khan S, Almeciga-Diaz CJ, Sawamoto K, Mackenzie WG, Theroux MC, Pizarro C, et al. Mucopolysaccharidosis IVA and glycosaminoglycans. Mol Genet Metab. 2017;120(1-2):78-95.

4. Sawamoto K, Alvarez Gonzalez JV, Piechnik M, Otero FJ, Couce ML, Suzuki Y, et al. Mucopolysaccharidosis IVA: Diagnosis, Treatment, and Management. Int J Mol Sci. 2020;21(4).

5. Tomatsu S, Averill LW, Sawamoto K, Mackenzie WG, Bober MB, Pizarro C, et al. Obstructive airway in Morquio A syndrome, the past, the present and the future. Mol Genet Metab. 2016;117(2):150-6.

6. Melbouci M, Mason RW, Suzuki Y, Fukao T, Orii T, Tomatsu S. Growth impairment in mucopolysaccharidoses. Mol Genet Metab. 2018;124(1):1-10.

7. Hendriksz CJ, Al-Jawad M, Berger KI, Hawley SM, Lawrence R, Mc Ardle C, et al. Clinical overview and treatment options for non-skeletal manifestations of mucopolysaccharidosis type IVA. J Inherit Metab Dis. 2013;36(2):309-22.

8. Khan SA, Peracha H, Ballhausen D, Wiesbauer A, Rohrbach M, Gautschi M, et al. Epidemiology of mucopolysaccharidoses. Mol Genet Metab. 2017;121(3):227-40.

9. Tomanin R, Karageorgos L, Zanetti A, Al-Sayed M, Bailey M, Miller N, et al. Mucopolysaccharidosis type VI (MPS VI) and molecular analysis: Review and classification of published variants in the ARSB gene. Hum Mutat. 2018;39(12):1788-802.

10. Giugliani R, Harmatz P, Wraith JE. Management guidelines for mucopolysaccharidosis VI. Pediatrics. 2007;120(2):405-18.

11. Berger KI, Fagondes SC, Giugliani R, Hardy KA, Lee KS, McArdle C, et al. Respiratory and sleep disorders in mucopolysaccharidosis. J Inherit Metab Dis. 2013;36(2):201-10.

12. Braunlin EA, Harmatz PR, Scarpa M, Furlanetto B, Kampmann C, Loehr JP, et al. Cardiac disease in patients with mucopolysaccharidosis: presentation, diagnosis and management. J Inherit Metab Dis. 2011;34(6):1183-97.

13. Alpoz AR, Coker M, Celen E, Ersin NK, Gokcen D, van Diggelenc OP, et al. The oral manifestations of Maroteaux-Lamy syndrome (mucopolysaccharidosis VI): a case report. Oral Surg Oral Med Oral Pathol Oral Radiol Endod. 2006;101(5):632-7.

14. Neufeld E. J. Muenzer in The Metabolic and Molecular Bases of Inherited Disease. McGraw Hill, New York; 1995.

15. Muenzer J. The mucopolysaccharidoses: a heterogeneous group of disorders with variable pediatric presentations. J Pediatr. 2004;144(5 Suppl):S27-34.

16. Tomatsu S, Montano AM, Oikawa H, Smith M, Barrera L, Chinen Y, et al. Mucopolysaccharidosis type IVA (Morquio A disease): clinical review and current treatment. Curr Pharm Biotechnol. 2011;12(6):931-45.

17. Hendriksz CJ, Berger KI, Giugliani R, Harmatz P, Kampmann C, Mackenzie WG, et al. International guidelines for the management and treatment of Morquio A syndrome. Am J Med Genet A. 2015;167A(1):11-25.

18. Hendriksz CJ, Harmatz P, Beck M, Jones S, Wood T, Lachman R, et al. Review of clinical presentation and diagnosis of mucopolysaccharidosis IVA. Mol Genet Metab. 2013;110(1-2):54-64.

19. Valayannopoulos V, Nicely H, Harmatz P, Turbeville S. Mucopolysaccharidosis VI. Orphanet J Rare Dis. 2010;5:5.

20. Baehner F, Schmiedeskamp C, Krummenauer F, Miebach E, Bajbouj M, Whybra C, et al. Cumulative incidence rates of the mucopolysaccharidoses in Germany. J Inherit Metab Dis. 2005;28(6):1011-7.

21. Malm G, Lund AM, Mansson JE, Heiberg A. Mucopolysaccharidoses in the Scandinavian countries: incidence and prevalence. Acta Paediatr. 2008;97(11):1577-81.

22. BioMarin. MPS IVa & VI: KSA Advisory Board. 2023 28/09/2023.

23. Moammar H, Cheriyan G, Mathew R, Al-Sannaa N. Incidence and patterns of inborn errors of metabolism in the Eastern Province of Saudi Arabia, 1983-2008. Ann Saudi Med. 2010;30(4):271-7.

24. Lehman TJ, Miller N, Norquist B, Underhill L, Keutzer J. Diagnosis of the mucopolysaccharidoses. Rheumatology (Oxford). 2011;50 Suppl 5:v41-8.

25. Wood T, Bodamer OA, Burin MG, D'Almeida V, Fietz M, Giugliani R, et al. Expert recommendations for the laboratory diagnosis of MPS VI. Mol Genet Metab. 2012;106(1):73-82.

26. Harmatz P, Shediac R. Mucopolysaccharidosis VI: pathophysiology, diagnosis and treatment. Front Biosci (Landmark Ed). 2017;22(3):385-406.

27. Kumar AB, Masi S, Ghomashchi F, Chennamaneni NK, Ito M, Scott CR, et al. Tandem Mass Spectrometry Has a Larger Analytical Range than Fluorescence Assays of Lysosomal Enzymes: Application to Newborn Screening and Diagnosis of Mucopolysaccharidoses Types II, IVA, and VI. Clin Chem. 2015;61(11):1363-71.

28. Akyol MU, Alden TD, Amartino H, Ashworth J, Belani K, Berger KI, et al. Recommendations for the management of MPS VI: systematic evidence- and consensus-based guidance. Orphanet J Rare Dis. 2019;14(1):118.

29. Giugliani R, Lampe C, Guffon N, Ketteridge D, Leao-Teles E, Wraith JE, et al. Natural history and galsulfase treatment in mucopolysaccharidosis VI (MPS VI, Maroteaux-Lamy syndrome)--10-year follow-up of patients who previously participated in an MPS VI Survey Study. Am J Med Genet A. 2014;164A(8):1953-64.

30. Schlander M, Beck M. Expensive drugs for rare disorders: to treat or not to treat? The case of enzyme replacement therapy for mucopolysaccharidosis VI. Curr Med Res Opin. 2009;25(5):1285-93.

31. Desnick RJ, Schuchman EH. Enzyme replacement and enhancement therapies: lessons from lysosomal disorders. Nat Rev Genet. 2002;3(12):954-66.

32. Pitz S, Ogun O, Arash L, Miebach E, Beck M. Does enzyme replacement therapy influence the ocular changes in type VI mucopolysaccharidosis? Graefes Arch Clin Exp Ophthalmol. 2009;247(7):975-80.

33. McGill JJ, Inwood AC, Coman DJ, Lipke ML, de Lore D, Swiedler SJ, et al. Enzyme replacement therapy for mucopolysaccharidosis VI from 8 weeks of age--a sibling control study. Clin Genet. 2010;77(5):492-8.

34. Crawley AC, Niedzielski KH, Isaac EL, Davey RC, Byers S, Hopwood JJ. Enzyme replacement therapy from birth in a feline model of mucopolysaccharidosis type VI. J Clin Invest. 1997;99(4):651-62.

35. Taylor M, Khan S, Stapleton M, Wang J, Chen J, Wynn R, et al. Hematopoietic Stem Cell Transplantation for Mucopolysaccharidoses: Past, Present, and Future. Biol Blood Marrow Transplant. 2019;25(7):e226-e46.

36. Penon-Portmann M, Blair DR, Harmatz P. Current and new therapies for mucopolysaccharidoses. Pediatr Neonatol. 2023;64 Suppl 1:S10-S7.

37. Bertolin J, Sanchez V, Ribera A, Jaen ML, Garcia M, Pujol A, et al. Treatment of skeletal and non-skeletal alterations of Mucopolysaccharidosis type IVA by AAV-mediated gene therapy. Nat Commun. 2021;12(1):5343.

38. Pierzynowska K, Gaffke L, Jankowska E, Rintz E, Witkowska J, Wieczerzak E, et al. Proteasome Composition and Activity Changes in Cultured Fibroblasts Derived From Mucopolysaccharidoses Patients and Their Modulation by Genistein. Front Cell Dev Biol. 2020;8:540726.

39. Zabinska M, Gaffke L, Bielanska P, Podlacha M, Rintz E, Cyske Z, et al. Decreased Levels of Chaperones in Mucopolysaccharidoses and Their Elevation as a Putative Auxiliary Therapeutic Approach. Pharmaceutics. 2023;15(2).

40. Harris JM, Martin NE, Modi M. Pegylation: a novel process for modifying pharmacokinetics. Clin Pharmacokinet. 2001;40(7):539-51.

41. Concolino D, Deodato F, Parini R. Enzyme replacement therapy: efficacy and limitations. Ital J Pediatr. 2018;44(Suppl 2):120.

42. Kida S, Koshimura Y, Yoden E, Yoshioka A, Morimoto H, Imakiire A, et al. Enzyme replacement with transferrin receptor-targeted alpha-L-iduronidase rescues brain pathology in mucopolysaccharidosis I mice. Mol Ther Methods Clin Dev. 2023;29:439-49.

43. Schaible P. Modifying enzyme replacement therapy - A perspective. J Cell Mol Med. 2023;27(2):165-73.

44. Zapolnik P, Pyrkosz A. Gene Therapy for Mucopolysaccharidosis Type II-A Review of the Current Possibilities. Int J Mol Sci. 2021;22(11).

45. Parenti G. Treating lysosomal storage diseases with pharmacological chaperones: from concept to clinics. EMBO Mol Med. 2009;1(5):268-79.

46. Kubaski F, de Oliveira Poswar F, Michelin-Tirelli K, Burin MG, Rojas-Malaga D, Brusius-Facchin AC, et al. Diagnosis of Mucopolysaccharidoses. Diagnostics (Basel). 2020;10(3).

47. Colmenares-Bonilla D, Colin-Gonzalez C, Gonzalez-Segoviano A, Esquivel Garcia E, Vela-Huerta MM, Lopez-Gomez FG. Diagnosis of Mucopolysaccharidosis Based on History and Clinical Features: Evidence from the Bajio Region of Mexico. Cureus. 2018;10(11):e3617.

48. Fateen E, Ismail MF, El-Boghdady NA, Aglan M, Ibrahim M, Radwan A. Differential diagnosis of mucopolysaccharidosis and oligosaccharidosis of a sample of Egyptian children. Bulletin of Faculty of Pharmacy, Cairo University. 2018;56(2):213-7.

49. Wood TC, Harvey K, Beck M, Burin MG, Chien YH, Church HJ, et al. Diagnosing mucopolysaccharidosis IVA. J Inherit Metab Dis. 2013;36(2):293-307.

50. Tomatsu S, Fujii T, Fukushi M, Oguma T, Shimada T, Maeda M, et al. Newborn screening and diagnosis of mucopolysaccharidoses. Mol Genet Metab. 2013;110(1-2):42-53.

51. Tomatsu S, Montano AM, Nishioka T, Gutierrez MA, Pena OM, Tranda Firescu GG, et al. Mutation and polymorphism spectrum of the GALNS gene in mucopolysaccharidosis IVA (Morquio A). Hum Mutat. 2005;26(6):500-12.

52. Litjens T, Hopwood JJ. Mucopolysaccharidosis type VI: Structural and clinical implications of mutations in N-acetylgalactosamine-4-sulfatase. Hum Mutat. 2001;18(4):282-95.

53. Vairo F, Federhen A, Baldo G, Riegel M, Burin M, Leistner-Segal S, et al. Diagnostic and treatment strategies in mucopolysaccharidosis VI. Appl Clin Genet. 2015;8:245-55.

54. Morrone A, Caciotti A, Atwood R, Davidson K, Du C, Francis-Lyon P, et al. Morquio A syndrome-associated mutations: a review of alterations in the GALNS gene and a new locus-specific database. Hum Mutat. 2014;35(11):1271-9.

55. Jezela-Stanek A, Rozdzynska-Swiatkowska A, Kulpanovich A, Ciara E, Marucha J, Tylki-Szymanska A. Novel data on growth phenotype and causative genotypes in 29 patients with Morquio (Morquio-Brailsford) syndrome from Central-Eastern Europe. J Appl Genet. 2019;60(2):163-74.

56. Tamarozzi ER, Torrieri E, Semighini EP, Giuliatti S. In silico analysis of mutations occurring in the protein N-acetylgalactosamine-6-sulfatase (GALNS) and causing mucopolysaccharidosis IVA. Genet Mol Res. 2014;13(4):10025-34.

57. Leong HY, Abdul Azize NA, Chew HB, Keng WT, Thong MK, Mohd Khalid MKN, et al. Clinical, biochemical and genetic profiles of patients with mucopolysaccharidosis type IVA (Morquio A syndrome) in Malaysia: the first national natural history cohort study. Orphanet J Rare Dis. 2019;14(1):143.

58. Bravo H, Neto EC, Schulte J, Pereira J, Filho CS, Bittencourt F, et al. Investigation of newborns with abnormal results in a newborn screening program for four lysosomal storage diseases in Brazil. Mol Genet Metab Rep. 2017;12:92-7.

59. Donati MA, Pasquini E, Spada M, Polo G, Burlina A. Newborn screening in mucopolysaccharidoses. Ital J Pediatr. 2018;44(Suppl 2):126.

60. Civallero G, Michelin K, de Mari J, Viapiana M, Burin M, Coelho JC, et al. Twelve different enzyme assays on dried-blood filter paper samples for detection of patients with selected inherited lysosomal storage diseases. Clin Chim Acta. 2006;372(1-2):98-102.

61. Montano AM, Tomatsu S, Gottesman GS, Smith M, Orii T. International Morquio A Registry: clinical manifestation and natural course of Morquio A disease. J Inherit Metab Dis. 2007;30(2):165-74.

62. Aldenhoven M, Sakkers RJ, Boelens J, de Koning TJ, Wulffraat NM. Musculoskeletal manifestations of lysosomal storage disorders. Ann Rheum Dis. 2009;68(11):1659-65.

63. Akyol MU, Alden TD, Amartino H, Ashworth J, Belani K, Berger KI, et al. Recommendations for the management of MPS IVA: systematic evidence- and consensus-based guidance. Orphanet J Rare Dis. 2019;14(1):137.

64. Hendriksz CJ, Burton B, Fleming TR, Harmatz P, Hughes D, Jones SA, et al. Efficacy and safety of enzyme replacement therapy with BMN 110 (elosulfase alfa) for Morquio A syndrome (mucopolysaccharidosis IVA): a phase 3 randomised placebo-controlled study. J Inherit Metab Dis. 2014;37(6):979-90.

65. Harmatz PR, Mengel E, Geberhiwot T, Muschol N, Hendriksz CJ, Burton BK, et al. Impact of elosulfase alfa in patients with morquio A syndrome who have limited ambulation: An open-label, phase 2 study. Am J Med Genet A. 2017;173(2):375-83.

66. Hughes D, Giugliani R, Guffon N, Jones SA, Mengel KE, Parini R, et al. Clinical outcomes in a subpopulation of adults with Morquio A syndrome: results from a long-term extension study of elosulfase alfa. Orphanet J Rare Dis. 2017;12(1):98.

67. Lin HY, Chuang CK, Ke YY, Hsu CC, Chiu PC, Niu DM, et al. Long-term effects of enzyme replacement therapy for Taiwanese patients with mucopolysaccharidosis IVA. Pediatr Neonatol. 2019;60(3):342-3.

68. Jones SA, Bialer M, Parini R, Martin K, Wang H, Yang K, et al. Safety and clinical activity of elosulfase alfa in pediatric patients with Morquio A syndrome (mucopolysaccharidosis IVA) less than 5 y. Pediatr Res. 2015;78(6):717-22.

69. Harmatz P, Ketteridge D, Giugliani R, Guffon N, Teles EL, Miranda MC, et al. Direct comparison of measures of endurance, mobility, and joint function during enzyme-replacement therapy of mucopolysaccharidosis VI (Maroteaux-Lamy syndrome): results after 48 weeks in a phase 2 open-label clinical study of recombinant human N-acetylgalactosamine 4-sulfatase. Pediatrics. 2005;115(6):e681-9.

70. Harmatz P, Giugliani R, IV DS, Guffon N, Teles EL, Miranda MCS, et al. Long-term follow-up of endurance and safety outcomes during enzyme replacement therapy for mucopolysaccharidosis VI: Final results of three clinical studies of recombinant human N-acetylgalactosamine 4-sulfatase. Mol Genet Metab. 2008;94(4):469-75.

71. Harmatz P, Giugliani R, Schwartz I, Guffon N, Teles EL, Miranda MC, et al. Enzyme replacement therapy for mucopolysaccharidosis VI: a phase 3, randomized, double-blind, placebo-controlled, multinational study of recombinant human N-acetylgalactosamine 4-sulfatase (recombinant human arylsulfatase B or rhASB) and follow-on, open-label extension study. J Pediatr. 2006;148(4):533-9.

72. But WM, Wong MY, Chow JC, Chan WK, Ko WT, Wu SP, et al. Enzyme replacement therapy for mucopolysaccharidosis VI (Maroteaux-Lamy syndrome): experience in Hong Kong. Hong Kong Med J. 2011;17(4):317-24.

73. Decker C, Yu ZF, Giugliani R, Schwartz IV, Guffon N, Teles EL, et al. Enzyme replacement therapy for mucopolysaccharidosis VI: Growth and pubertal development in patients treated with recombinant human N-acetylgalactosamine 4-sulfatase. J Pediatr Rehabil Med. 2010;3(2):89-100.

74. Harmatz P, Hendriksz CJ, Lampe C, McGill JJ, Parini R, Leao-Teles E, et al. The effect of galsulfase enzyme replacement therapy on the growth of patients with mucopolysaccharidosis VI (Maroteaux-Lamy syndrome). Mol Genet Metab. 2017;122(1-2):107-12.

75. Braunlin E, Rosenfeld H, Kampmann C, Johnson J, Beck M, Giugliani R, et al. Enzyme replacement therapy for mucopolysaccharidosis VI: long-term cardiac effects of galsulfase (Naglazyme(R)) therapy. J Inherit Metab Dis. 2013;36(2):385-94.

76. Brands MM, Oussoren E, Ruijter GJ, Vollebregt AA, van den Hout HM, Joosten KF, et al. Up to five years experience with 11 mucopolysaccharidosis type VI patients. Mol Genet Metab. 2013;109(1):70-6.

77. Horovitz DDG, Acosta AX, de Rosso Giuliani L, Ribeiro EM. Mucopolysaccharidosis type VI on enzyme replacement therapy since infancy: Six years follow-up of four children. Mol Genet Metab Rep. 2015;5:19-25.

78. Concolino D, Amico L, Cappellini MD, Cassinerio E, Conti M, Donati MA, et al. Home infusion program with enzyme replacement therapy for Fabry disease: The experience of a large Italian collaborative group. Mol Genet Metab Rep. 2017;12:85-91.

79. Milligan A, Hughes D, Goodwin S, Richfield L, Mehta A. Intravenous enzyme replacement therapy: better in home or hospital? Br J Nurs. 2006;15(6):330-3.

**Tables**

Table 1: Consensus Statements – Screening and Diagnosis

| Consensus Statement | Agreement Level (%) |
| --- | --- |
| There is a need to enhance the availability of diagnostic and monitoring tests for MPS IVa and VI across treatment centers in Saudi Arabia, including urinary GAG tests, enzyme activity assays, and DBS tests. | 100 |
| Genetic counseling and molecular genetic testing should be made available to all individuals with suspected MPS IVa and VI. | 100 |
| Among select families with a history of MPS IVa and/or MPS VI in Saudi Arabia, premarital screening initiatives should be encouraged to improve primary prevention of disease cases. | 100 |
| A newborn screening pilot initiative utilizing LC-MS/MS should be established to determine the incidences of these disorders in newborns and to improve secondary prevention of MPS IVa and VI in Saudi Arabia. | 100 |
| There is a need to raise awareness of LSDs among healthcare professionals of different subspecialties in the country to improve the detection of disease cases. | 100 |
| Community-based awareness initiatives should be conducted to combat sociocultural issues like stigmatization and isolation. | 100 |

DBS, dry blood spot; GAG, glycosaminoglycan; LC-MS/MS, liquid chromatography/tandem mass spectrometry; LSD, lysosomal storage disease; MPS, mucopolysaccharidoses.

Table 2: Consensus Statements – Management

| Consensus Statement | Agreement Level (%) |
| --- | --- |
| It is recommended that treatment for MPS IVa and VI is initiated immediately after confirmation of diagnosis and family counseling to discuss treatment options and expected outcomes. | 100 |
| ERT (elosulfase alfa for MPS IVa and galsulfase for MPS VI) should be accessible to all eligible patients in Saudi Arabia. | 100 |
| Formal standards of care for MPS IVa and VI should be established in Saudi Arabia, encompassing clear guidelines that specify when ERT is indicated as a viable treatment option and when treatment cessation is warranted. | 100 |
| ERT home infusion programs should be developed along with clear selection criteria and guidelines to improve adherence among patients. | 100 |
| Utilizing multidisciplinary care teams that include metabolic specialists, surgeons, nurses, physiotherapists, occupational therapists, psychologists, audiologists, and speech pathologists will enhance patient outcomes and alleviate disease burden associated with MPS IVa and VI. To address the multisystemic manifestations of these conditions, care teams should also engage pulmonology, dental, orthopedic, pediatric, ENT, ophthalmology, cardiology, anesthesiology, and neurology specialists as required. | 100 |
| As part of the integrated, comprehensive care approach, patients should receive extensive psychological support and education to address issues such as stigmatization and isolation. | 100 |
| There is a pressing need to increase the number of specialized adult geneticists in the country. | 100 |
| In all cases, treatment plans in MPS IVa and VI should be highly individualized. Informed decision-making should be promoted with extensive patient/caregiver counseling, as well as discussion of treatment options and their benefits and limitations by or in close consultation with a genetic/metabolic specialist to address the specific needs and clinical profile of each patient. | 100 |
| There is a need to establish national centers of excellence for the management of LSDs in Saudi Arabia to ensure the delivery of standardized, comprehensive care. | 86 |
| Healthcare staff involved in the delivery of care for MPS IVa and VI should undergo periodic training and education to stay updated on the latest advancements in emerging therapies and management guidelines. | 100 |
| It is crucial to establish robust systems for monitoring and efficiently integrating approved advanced therapies and combination therapies for MPS IVa and VI within the healthcare system of Saudi Arabia. | 100 |
| It is recommended that regional experts in collaboration with the appropriate national health authorities publish updated guidelines pertaining to the management of MPS IVa and VI. The guidelines should include standards of care, protocols for diagnosis and treatment, and directives pertaining to the establishment of multidisciplinary care teams and centers of excellence. | 100 |
| There is a need to establish a national reimbursement program for rare diseases in Saudi Arabia to improve access to crucial medications and care. This program should also address access for the expatriate subpopulation. | 100 |

ENT, ear, nose, and throat; ERT, enzyme replacement therapy; LSD, lysosomal storage disease; MPS, mucopolysaccharidoses.

Table 3: Consensus Statements – Data Generation and Reporting

| Consensus Statement | Agreement Level (%) |
| --- | --- |
| There is a pressing unmet need for local MPS IVa and MPS VI data generation and the establishment of a centralized, robust national registry for LSDs. | 100 |
| There is a critical need for enhanced collaboration and data sharing among treatment centers in Saudi Arabia. | 100 |

LSD, lysosomal storage disease; MPS, mucopolysaccharidoses.

**Figure Legends**

Figure 1: Level of agreement with the statement “HSCT is ineffective in MPS IVa and is not recommended for these patients” as per a panel of consultant geneticists in Saudi Arabia (number of respondents = 6) (22).

HSCT, hematopoietic stem cell transplantation; MPS, mucopolysaccharidoses.

Figure 2: Proportion of patients with MPS IVa or MPS VI that are expatriates as per a panel of consultant geneticists in Saudi Arabia (number of respondents = 6) (22).

MPS, mucopolysaccharidoses.
