# Supplementary material for: Consensus-based expert recommendations on the management of MPS IVa and VI in Saudi Arabia
Source: Orphanet J Rare Dis. 2024 Jul 17;19:269. doi: 10.1186/s13023-024-03237-3 (PMC11253461; doi:10.1186/s13023-024-03237-3)
Supplement: Supplementary file 1 — Supplementary Material 1 [file 13023_2024_3237_MOESM1_ESM.docx]

Reviewer reports:

Reviewer #1: I would like to thank the invitation to review the original manuscript entitled "Consensus-based expert recommendations on the management of MPS IVa and VI in Saudi Arabia", submitted to Orphanet Journal of Rare Diseases. This is an important original manuscript presenting the main recommendations brought by experts involved in the care of patients with MPS types IVa and VI and researchers. This study has a lot of importance for patients and their caregivers, and this must be certainly a topic of interest to all healthcare professionals. The content is organized and easy to be understood by different specialties. Consensus statements regarding management and therapies disclosed in Table 2 emphasize on the main needs presented by most MPS patients and also highlights several important aspects related to multidisciplinary care. The important idea of proposing a national registry for lysosomal storage diseases will be certainly one of the most significant purposes presented in this consensus. Some points could be briefly evaluated by the authors at this time of review:
1. The introduction of the consensus paper is a lot interesting and looks like a mini review, however it is large for its purpose (total: 138 lines). Maybe some aspects could be summarized by the authors, such as the excerpt between lines 103 and 106, which could be suppressed.
2. One important aspect which could be implemented by the authors is a comparison chart, presenting, for example, the main consensus obtained from the current study and compare it with previous recommendations, consensus and guidelines related to MPS type VI or MPS type IVA.

**Thank you for your insightful comments on our consensus paper. Indeed, our recommendation to establish a comprehensive national registry for lysosomal storage diseases could be highly significant in the context of MPS management in Saudi Arabia.**

**As per your suggestion, we have condensed some of the content in the ‘Background’ section by removing some sentences. The section is now more concise and has a better flow. We amended lines 103-106, and this part is now combined with the previous paragraph.**

**Regarding the very interesting point of comparing the statements with previous recommendations, we have now added in a couple of places in the ‘Discussion’ section where the consensus statement discussed aligns with previously published recommendations (pertaining to multidisciplinary care and immediate treatment initiation after diagnosis). However, as our other statements are specific to Saudi Arabia, they pertain to topics important in our country (such as premarital screening, newborn screening, accessibility and coverage, national registry, etc.). For these statements, there is not much overlap with previously published recommendations, and therefore it would be difficult to create a comprehensive comparison chart for that purpose.**

**Thank you once again for your valuable comments. They have helped improve the quality and readability of our manuscript.**

Reviewer #2: The manuscript lists the recommendations of seven specialist doctors from Saudi Arabia to test, diagnose, treat and report MPS IVa and VI. These are diseases with a special incidence in Saudi Arabia. These recommendations have been reached by total consensus of the seven specialists.
The article does not present any data that helps to better understand these diseases from a genetic, biochemical or physiological point of view.
It seems logical to think that doctors specialized in these diseases are the ones who best know how to treat and prevent them. Furthermore, being from Saudi Arabia, they are the ones who best know how the health system is organized and the social, economic and cultural situation of the country. For all these reasons, I think that these recommendations can help in the fight against MPS IVa and VI in Saudi Arabia.

**Thank you for the valuable comments on our consensus manuscript. Indeed, these recommendations are specific to Saudi Arabia and are based on the local population and healthcare system. Our aim for this publication is to promote consistent care for these rare diseases and help optimize the patient pathway in the country. Thank you once again for your valuable comments.**
